# Child-directed speech is optimized for syntax-free semantic inference

**DOI:** 10.1038/s41598-021-95392-x

**Published:** 2021-08-16

**Authors:** Guanghao You, Balthasar Bickel, Moritz M. Daum, Sabine Stoll

**Affiliations:** 1 Department of Comparative Language Science, University of Zurich, 8050 Zurich, Switzerland; 2 Center for the Interdisciplinary Study of Language Evolution (ISLE), University of Zurich, 8050 Zurich, Switzerland; 3 Department of Psychology, University of Zurich, 8050 Zurich, Switzerland; 4 Jacobs Center for Productive Youth Development, University of Zurich, 8050 Zurich, Switzerland

**Keywords:** Human behaviour, Statistical methods

## Abstract

The way infants learn language is a highly complex adaptive behavior. This behavior chiefly relies on the ability to extract information from the speech they hear and combine it with information from the external environment. Most theories assume that this ability critically hinges on the recognition of at least some syntactic structure. Here, we show that child-directed speech allows for semantic inference without relying on explicit structural information. We simulate the process of semantic inference with machine learning applied to large text collections of two different types of speech, child-directed speech versus adult-directed speech. Taking the core meaning of causality as a test case, we find that in child-directed speech causal meaning can be successfully inferred from simple co-occurrences of neighboring words. By contrast, semantic inference in adult-directed speech fundamentally requires additional access to syntactic structure. These results suggest that child-directed speech is ideally shaped for a learner who has not yet mastered syntactic structure.

## Introduction

Understanding the meaning of words is one of the cornerstones of language learning. Learners need to cope with the meaning of many thousands of words with intricate meaning nuances and connotations across many different contexts. The basic mechanisms in this endeavor rely on cognitive abilities such as the categorization of objects and events^1–6^, and socio-cognitive abilities such as gaze following, joint attention, and gesture interpretation^7–11^. Meaning of words can be understood through cross-situational learning, relating the words with their extra-linguistic referents occurring in various situations^12–14^.

However, words and their meanings rarely occur in isolation^15,16^ and this obscures simple matching of sound patterns with perceived objects or events in the world. In most cases, understanding what a word means requires understanding its surrounding words in an utterance—or as the linguist John Rupert Firth put it over 70 years ago: “You shall know a word by the company it keeps”. In fact, word co-occurrences have been shown to reflect semantic associations of words, thus efficiently capturing intricate word meanings^17,18^. Learning these associations requires a highly efficient machinery of distributional learning, which is a fundamental learning mechanism that relies on statistical distributions of elements in the linguistic and extra-linguistic environment^13,19–24^. Children use statistical distributions from the speech they hear for inferring various phonological^25–28^ and grammatical generalizations^29–33^.

For learning meaning, however, most current theories agree that distributional information alone is not sufficient. For example, the theory of “syntactic bootstrapping” proposes that children critically rely on syntax to infer meaning^34^. Support for this proposal comes from evidence that children rely on syntactic frames of argument structure to elicit causative meanings^35^. It has been generally posited that children recognize that words are not simply serialized like beads on a string, but that they are part of compositional syntactic structures, either by drawing on abstract innate rules^36,37^ or by detecting item-specific constructions^38,39^. Indeed, the compositional syntax of language has been posited as a property that is so fundamental for language that its recognition demarcates human learners from other animals^40^, even if the underlying processing machinery may become fully mature only relatively late in brain ontogeny^41^.

Here, we test this proposal in the example of causation as a key dimension of meaning in human language and cognition^42–44^ and concept formation in language learning^45–47^. Causation is one of the cornerstones of event conceptualization and one of the main topics in conversation (“Who did it?”, “Why did it break?”). But how can infants learn the general meaning of causation? In a sentence with a causal clause such as “The vase broke because it fell down”, such inference is facilitated by the explicit conjunction “because”. Causation can also be openly marked by fixed constructions as in “She had her assistant type the report”. Here the verb “had” indicates the causation meaning of the utterance. However, causation is often only implicitly entailed. For example, in “I broke the window”, there is no dedicated marker that signals my actions as the cause of the breaking event. Given the absence of a formal linguistic marker in most causal verbs in English, how can infants infer the common meaning of causation in words like *break, kill, enable, lead, bend* etc. and thereby learn to understand the causal structure of the events involved? And what cues from the linguistic context can children rely on in this process?

In line with received theory, previous research suggests a critical role of syntactic structures for the understanding of causative concepts. For example, a sentence like “He broke the vase” comes with a syntactic structure of the causative verb “break”, which contains a subject and an object. Concretely, the structure assigns “he” the role of the subject of the sentence in an active voice sentence, while labeling “the vase” as the object. This in turn invites the interpretation of “he” as the causer and “the vase” as the causee of the action “break”. Accordingly, the recognition of such structures may help infants narrow down verb meanings. This theory is supported by experimental evidence that children can successfully detect structural cues in the input (such as word order or grammatical markers) to infer the relevant syntactic frames, either in the abstract^34,35,48–53^ or with more concrete items^54,55^, and to extract meanings from these frames. As this ability has been found in children at age 2 or even earlier, it is plausible that it plays a critical role outside experimental settings as well.

However, the input that children receive in natural environments may contain so much richer statistical information that cue-based inferences of syntactic structures might not be needed when children learn causative concepts. For example, hearing a sentence like “You will hurt yourself if you break the glass”, the word “break” is probabilistically associated with the meaning of the subsequent expression “the glass” already without knowing that “the glass” is the object of “break” in syntax. In addition, the verb “hurt” provides not only thematic information but also indicates the result caused by “break”, again regardless of the syntax. As the range of contexts expands, further thematically-related items become relevant, indicating, for example, the difference between a physical and a social change of state (“break the glass” vs. “break the rule”). In an extreme scenario, if presented only the list of words “hurt”, “break” and “glass”, one could still possibly make the same inference with no syntax at all. Hence, even without recognizing syntax, the causative meaning of “break” will be understood differently from non-causative meanings. In fact, it has been recently found that reduced syntax does not hinder us from communicating with Broca’s aphasics^56^ or in a language with simple grammar^57^, as inference is possible from co-occurring words^58,59^.

To assess whether such contextual information is indeed sufficient to learn meaning, we employed a computational method that systematically tests and compares different sets of information in natural speech. We thereby automated the process of semantic inference with computational power, grounded by the theory of distributional learning. We ask two questions. First, does the range of available information about causal semantics via distributional learning differ in child-directed speech from other speech genres? Second, is the raw immediate semantic context (the meaning of surrounding words in the same utterance) indeed already sufficient, or does a learner need to recognize and use syntactic structures for successful inference of causatives? Among many sources of linguistic input, child-directed speech is the primary one where children are immersed. Both the amount and the quality of child-directed speech have been shown to directly influence children’s language development^60–65^. In addition, most theories emphasize that child-directed speech differs from adult conversation both in terms of structure, lexical richness, and intonation^66^, potentially facilitating language learning^67,68^. We therefore compare distinctiveness of the information distribution between the genres child-directed speech and adult conversation and, as a control with more complex structure, written language.

We used transcribed and annotated speech of these three different genres, collected in what is technically known as corpora. To obtain insight into learning opportunities from real-life input, we simulated the human parser in testing for syntactic and semantic information. We tested different manipulations of semantic and syntactic information in the speech addressed to 14 English learning children from the Manchester corpus^69,70^ recorded in natural interactions, adult conversation from the spoken part of the British National Corpus (BNC)^71^, and written language from the written part of the BNC. Each corpus was sampled by recording session to reach an approximately equal number of tokens of ca. 3.2 million each. Every sentence was parsed into lexical words (lemmas), tagged for word class and annotated for grammatical dependencies with spaCy^72^, a standard natural language processing toolkit.

We applied a distributional learning algorithm, namely word embeddings (Word2Vec) with an adjustable window^17^. This window is a means of controlling the length of text around the target word that serves as the search space. The algorithm is based on co-occurrence features and therefore can be used to examine which features of surrounding words help in category generalization of a target word.

We evaluated the quality of causative inference in each model by examining the discrimination it achieves between selected sets of causatives (23 words) and non-causatives (9 words)^73,74^. We measured discrimination success by the extent to which the obtained pairwise cosine distance between causative and non-causative meanings exceeds baseline distances between randomly selected words.

## Results

**Figure 1:**
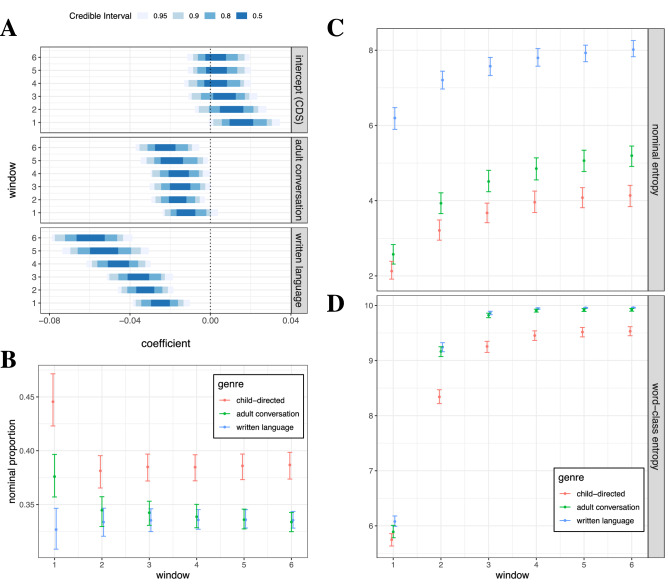
Causative meanings are better discriminated than what is expected by chance in child-directed speech (CDS) but not in adult conversation and written language, regardless of how many neighboring words are included in a window (**A**). Credible intervals represent highest posterior densities. A likely cause of this is increased repetitiveness of the contexts around verbs (**B**–**D**, all shown with bootstrapped error estimates): Child-directed speech shows a higher proportion of nominals surrounding verbs, especially with the smallest window (**B**); These nominals around verbs are more repetitive in child directed-speech than in the other genres, as measured by Shannon entropy (**C**); Verbs are surrounded by a less varied range of word-class constructions in child-directed speech as measured by Shannon entropy (**D**). Both entropies increase with larger windows due to richer samples, which corresponds to the decrease in learning performance (**A**).

In Study 1, we tested for a genre difference in causative discrimination of raw utterances. We regressed the above-baseline distance on speech genre using a hierarchical Bayesian model (Fig. 1A). Child-directed speech was set as the reference level of speech genre. Individual verbs (both causatives and non-causatives) were treated as group-level factors, with both random intercepts and slopes, so that biases in verb selection were controlled.

The results show that the cosine distance exhibited by child-directed speech largely shifts from the baseline to the positive when the window is small, suggesting above-chance discrimination of causative meanings. In particular, with window size 1, the cosine distance significantly surpasses the baseline (96.6% of the posterior samples are above 0, see Fig. 1A). By contrast, both adult conversation and written language lead to substantially less discriminative power than child-directed speech, especially when the window size increases. Predicted cosine distances are all close to or below the baseline (see Fig. S1 in Supplementary information). Leave-one-out cross-validation^75,76^ reveals that models with genre as a predictor leverage almost all of the total weight in predictive performance (see Fig. S2 in Supplementary information). In order to examine what aspects of semantics might be learned, we performed clustering analysis for the model with window size 1 in child-directed speech using neighbor joining^77^ (see Sect. S1 and Figs. S3 and S4 in Supplementary information). Prototypical causatives, together with other words with causal semantics, can fall under the same cluster (see the example cluster in Fig. S4), further suggesting that the specific semantic domain of causatives can be inferred from raw utterances.

A likely reason for the increased discriminative power and the inference of causal semantics in child-directed speech is the extensive involvement of nominals and the repetitive nature of the elements that surround each verb. Child-directed speech shows a higher proportion of nominals around verbs across all windows than the other genres, especially within the smallest window size (Fig. 1B) where performance is best (Fig. 1A). These nominals, at the same time, display much less variation in child-directed speech, thus suggesting higher repetitiveness (as measured by the Shannon entropy per window size; Fig. 1C). Furthermore, the words around the verb vary much less in their word class in child-directed speech than in the other genres (Fig. 1D). For instance, for the verb “open”, we find a high proportion of the string “PRONOUN open PRONOUN” in child-directed speech (77 out of 974 uses of “open” as a verb), as well as a number of occurrences of “PRONOUN open DET NOUN”, such as “shall I open the lid?” or “why don’t you open the tin?”. Other causative meanings occur in very similar frames (e.g., “break” as in “mummy would break the hat” and “don’t break the doll”, or “turn” as in “turn the knob” and “shall I turn the page?”). The repetitive nature and frequency of such frames are known to critically help learn categories^31,78^. Here, the causative category can be learned from a frequent frame that describes an agent acting on an patient undergoing change.

**Figure 2:**
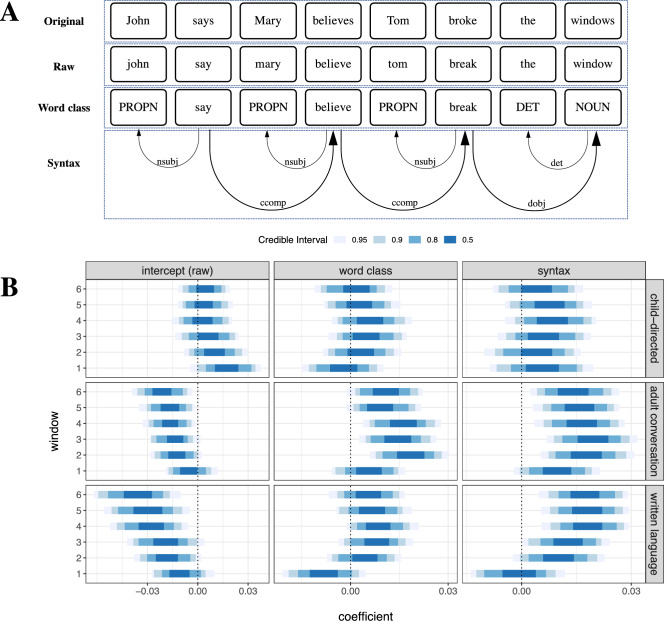
“Raw” gives the lexical entries of the original text, “word class” shows the word-class tags surrounding the verbs, and “syntax” denotes the dependencies that the verbs open (in the CLEAR style^90^ as tagged by spaCy). The dependencies are represented by arrows with the relation tags on top. The training data for each layer (word class vs. syntax) consists of both the raw utterances and the information from the respective layer (**A**). Discrimination of causative meanings remains stable or deteriorates when further syntactic information is made available in child-directed speech, but mostly improves when such information is made available in adult conversation and written language (**B**). Credible intervals represent highest posterior densities.

In Study 2, we assessed the extent to which additional structural information improves the identification of causative meanings. Specifically, we tested whether access to word class information (tags like “noun” or “verb”) or syntax (in the form of dependency relations) improves causative discrimination beyond the baseline performance on raw text (Fig. 2A). Our models again include individual verbs as group-level factors (both random intercepts and slopes). Results show that in child-directed speech, the additional information does not improve causative inference (Fig. 2B; across window sizes, 0 is included in all 90% CIs and almost all 80% CIs). In other words, the frames from which meaning is inferred need not contain any information beyond word co-occurrences. Also, models with additional information on word class and dependencies have considerably worse predictive performance in cross-validation than models without this information (Fig. S2 in Supplementary information).

This is strikingly different with adult conversation and written language. Here, models achieve above-baseline causative discrimination only with access to additional information. As already noted in Study 1, raw text alone yields worse-than-baseline discrimination, especially when window sizes increase. Causative inference is possible only with access to word class or syntax information. In particular, syntax has a consistent impact in both adult-directed genres except with window size 1, while word classes enhance the performance only in adult conversation and show only marginal improvements in written language. Models with additional syntactic information leverage considerably more weight in predictive performance than in child-directed speech (except again in window size 1; see Fig. S2 in Supplementary information). A likely reason for the exceptional pattern in window size 1 is that short windows contain only insufficient cues from syntactic information. Larger windows contain richer syntactic information from which the learning algorithm profits.

## Discussion

Our results suggest that child-directed speech is ideally suited to a statistical learner who has little access to structural information but excels in recognizing, memorizing and generalizing patterns from frequently repeated patterns in raw input. This ability is a critical endowment of infants that has been experimentally demonstrated for the learning of various sound patterns^24,66,79–81^. By using a distributional learning algorithm to thoroughly examine words with their respective contexts at the utterance level, we predict that when learning meaning, children may leverage the statistical idiosyncrasies in child-directed speech, including the repetitive nature and the excessive involvement of nominals that we discovered. As the word embeddings algorithm neutrally examines all the co-occurrence patterns (not limited to patterns accompanying verbs) in the corpus, our results may allude to the general characteristics such as simplicity and repetitiveness in child-directed speech, which can facilitate semantic inference.

Such facilitation of raw input of child-directed speech might be relevant to the typical co-occurring words in transitive frames in English for causatives. However, the window-size-one frame of raw text does not seem to help with the causative discrimination in adult conversation and written language; neither do the frames of size two and three help, although they are also likely to capture the typical transitivity. This indicates that compared to child-directed speech, raw utterances of adult conversation and written language may generally contain more diversely distributed information surrounding causative verbs, thus supplying less explicit transitive or causative frames to support semantic inference. Distributional learning in these two genres significantly profits from additional structural information. This indicates that syntax may differ across the speech genres. While adult conversation and written language may contain rich structural information, syntax might stay rudimentary in child-directed speech and therefore exerts little additional impact on meaning discrimination. This again resonates with our findings in Study 1, where raw input without structural information suffices in semantic inference, and it is consistent with neurobiological evidence that the syntactic processing system matures only quite late in childhood^41^, at an age where children have fully acquired the meaning of causative verbs^82^.

It should be noted that the raw utterances in our studies are maximally syntax-free when processed by the model, even excluding the information of word order (the Word2Vec algorithm dissolves the order of contextual words in its predictive training; see more detail in Methods). In other words, the raw learning materials are simple word co-occurrences that entail no explicit syntactic information of any kind. Hence, the causal inference via statistical learning in our results of the “raw” layer can be fully accounted for by syntax-free information as the learning resources. Although the syntax-free condition is of theoretical interest and causative semantics indeed emerges from this learning condition, word order may be considered so fundamental in language learning (and processing) that disregarding it may seem unnatural. Our goal here, however, was to test whether generalizing causative semantics is even possible without presupposing knowledge about word order. To simulate a learner who attends to order, future studies may incorporate word order in the near-syntax-free training of raw utterances. Here, models that are sensitive to sequential information (e.g., BERT^83^) would be useful. At the same time, however, such models require long sequences with closed contexts and may not realistically mirror the short individual chunks characteristic of child input and rapid conversation.

We end by pointing out two limitations of our studies. First, while we simulate possible mechanisms, we do not directly test how children actually perceive and process the raw utterances. It has been shown that syntactic knowledge may emerge from raw input^31,33,78^, which can facilitate semantic inference^34,35^. As much as we recognize such emergence of syntax, it does not contradict our findings that raw input is sufficient for semantic inference, as our model is not informed of the potential syntactic knowledge. In fact, both semantic inference and syntactic abstraction, if any, can be the end products of distributional learning from raw input alone, and these two processes do not necessarily depend on each other. That said, when both processes take place extensively, there is likely close interaction between them, and the raw utterances and the syntactic information may work in synergy to render the best semantic inference, as suggested by our results for adult conversation and written language. Child-directed speech, by contrast, does not benefit from this synergy, at least with the word-class and dependency syntax that we take into account. At the very least, our findings clearly speak to the distinction between child-directed speech and the other speech genres. Distributional information is uniquely shaped in child-directed speech, which is an important facilitator for young children to learn word meanings.

Second, by taking English, an analytic language, as a test case, we only focus on distributional context at the word level in the present research. There has been evidence that learning from contextual frames universally relies on items at the morpheme level instead of the word level^78^. Also, for morphologically complex languages such as Turkish, previous studies have revealed the importance of verbal morphology and case marking for language comprehension, especially for understanding causal meaning^84–86^. Hence, to generalize our findings to typologically diverse languages, future research should look at semantic learning from distributional information in raw input at both word and morpheme levels.

## Methods

### Corpora

We used three corpora as the training source for the genres child-directed speech, adult conversation, and written language. As corpus for child-directed speech, we extracted the adult speech directed to the target children in the Manchester Child Language Corpus^69,70^. The 12 children in the Manchester Corpus^69^ are aged from 20 to 36 months, while the 2 children in the MPI-EVA-Manchester Corpus are intensively recorded when they are 2 and 4 years old. For the adult conversation corpus we extracted naturalistic conversations from the British National Corpus. The written corpus uses written texts from the British National Corpus^71^. The original size of these corpora vastly differs (Table S1 in Supplementary information). We therefore extracted subsets of the same size of the two other corpora to ensure the comparability of the three corpora.

Since the word embeddings algorithm iterates through the tokens in the corpus, we sampled the corpora to approximately an equal number of tokens, which was the maximum number of tokens we could obtain from the child-directed speech in the Manchester corpus. Thus, no sampling was involved in the Manchester corpus. Crucially in the sampling process for the other two genres, we took one session with all of its utterances at a time to maintain the coherence of semantics in our training sources. Otherwise, random sampling of utterances from all sessions might have included more infrequent words that lack deep inference. Table S2 in Supplementary information shows the summary of the sampled corpora.

### Data processing

We based our studies on the lemmatized utterances from the selected corpora. These lemmatized utterances formed the fundamental “raw” layer in the studies. Lemmatization was a necessary step in our approach to obtain sufficient occurrences of each word type to generate the embeddings. It was feasible for English, an analytic language, as the lemmas are minimally obscured by affixation.

We mainly tested for two layers of syntactic information, as shown in Fig. 2A, which included (i) word-class tags in layer “word class” and (ii) relation tags from dependencies in layer “syntax”. Besides, to probe how the semantic inference is impacted by the abstractness of syntactic information, we modeled an additional layer “lexicon” that included dependency structure in the form of lexical words, that is, syntax is represented implicitly by concrete words based on their dependency structure (see Sect. S2 and Fig. S5 in Supplementary information). These three layers were combined with the lemmatized utterances to form the sources for subsequent model training. Concretely, when fed to the algorithm, the training instances from the syntactic layers were shuffled with the the raw utterance, in order to simulate an unbiased order of processing semantics and syntax of each utterance^87–89^. Details are given as below.

#### Lemmatization and tagging

We applied the spaCy natural language processing toolkit^72^ for lemmatization, word-class tagging, and dependency tagging in the CLEAR style^90^ as tagged by spaCy. The main consideration behind this universal processing manner was a unified standard for all the comparisons to be conducted. The original lemmas and annotations, including word-class tagging and dependency tagging, employ different standards and naming conventions. For instance, lemmas of pronouns in the Manchester corpus keep their original forms instead of transforming to the hw (headword) as in the BNC (e.g., “him” being “him” in the Manchester corpus while being “he” in the BNC). Worse, no dependency information is available in BNC. We therefore used a universal processing tool to enhance the consistency of data forms across corpora and ensure the comparability of results in different corpora. Nonetheless, an additional test using existing hand-tagged annotations from the Manchester corpus was also conducted, so as to ensure that the effects of our main studies were not substantially affected by automatic parsing. Results of this additional test are reported in Sect. S3 and Fig. S6 in Supplementary information.

In all three syntactic layers, verbs were kept as their lemmas, since we were particularly interested in the categorization of verb meaning. Additionally, in order to maximally infer from the words and tags in dependencies, minimal dependency subtrees were extracted to strengthen the co-occurrence patterns potentially learned by the distributional algorithm. Every subtree consisted of a dependency head and its direct dependents, so the number of subtrees was determined by the number of dependency heads in one utterance. Table S3 in Supplementary information gives an example of subtrees extracted for the utterance in Fig. 2A.

#### Start and end of utterances

We added additional special symbols to mark the start and the end of each utterance. This was driven by the consideration of the major limitation of Word2Vec training on short utterances. For instance, when the algorithm encounters a one-word utterance, no co-occurrence pattern is to be inferred, but this utterance, if consisting of only a verb, could suggest semantic and syntactic features such as argument structure and imperative mood (e.g. “Run!” with the start and the end markers could be understood as an imperative and intransitive structure). Even worse, the proportion of one-word utterances is large in child-directed speech (Table S4 in Supplementary information), which might greatly influence the overall vectorization. Hence, we employed special symbols “$${}^{\wedge \wedge }$$” and “$$” to mark the start and the end of each utterance respectively. The layer of word class also included this marking to reinforce the learning. On the other hand, minimal dependency subtrees were fragmented and extracted from each full utterance; therefore, this additional marking was not employed in the layers “syntax” and “lexicon”.

The training of context was thus at the utterance level bound by the start and the end, without being extended to the context in discourse. Although the broader sense of context in discourse can be beneficial, it remains problematic to determine what turns in discourse are relevant to the current utterance. Besides, the scope of syntactic information is limited within each utterance. We therefore only focused on context in each utterance in the present research.

### Training with Word2Vec

#### Word embeddings

We used the original word embeddings model proposed by Mikolov et al.^17^ in our studies. This model is based on simple word co-occurrences and can be easily built from scratch computationally, which suits our objectives of simulating distributional learning. Concretely, word embedding algorithms resemble human distributional learning by building connections between contextual frames and target words, and are able to represent word meaning in high-dimensional space. Compared to more complex models such as the transformer model BERT^83^, which achieves desirable performance with attention over long sequences (even by concatenating short sequences^91^), Word2Vec models do not rely on long utterances, which are rare in child-directed speech: the mean length of utterances is 4.19 (Table S4 in the Supplementary Information) and concatenating utterances would be problematic due to non-adjacent or often distant turns of child-directed speech in the corpora. Instead of using sequential information, the Word2Vec models exploit an adjustable model to determine the range of contexts. Such a window includes text from both sides of the target word, e.g., when the window size is 1, it includes 3 words, the target word in the middle and one word on each side. With each window of word co-occurrences, the algorithm employs a neural network to predict the central unit from the context, or vice verse, where the order of contextual words is dissolved (i.e., all contextual words are treated equally regardless of their position in the context). This is crucial for excluding word order as a potential syntactic factor involved in training. Suppressing word order maximally ensures that no structural information of any kind is involved in the training of raw utterances, thus simulating a syntax-free learning condition. Moreover, we intended to generate one vector for each word type and calculate the overall distances between verb types, for which the original Word2Vec is better suited than the contextualized token-level embeddings of BERT^83^ and similar models.

Mikolov et al.^17^ propose two models within the framework of word embeddings—continuous bag of words (CBOW) and skip-gram (SG). While CBOW employs an averaging step where the semantics of context words is fused, SG better preserves the feature of each individual context word without averaging. In addition, SG yielded better performance in the original word similarity tasks^17^. We therefore chose SG as the method for our semantic representation.

We utilized the word embeddings toolkit developed by Rehurek and Sojka^92^. We set the dimension of vectors to 200 and the number of iteration to 100 to generate robust embeddings, and this setup was the same for all models to ensure the comparability of the semantic representations. Higher dimension and iteration can help capture word semantics more effectively^93^ (see Fig. S7 in Supplementary information for a test of lower dimensionality which can reduce the quality of embeddings). The loss in the training progress is shown in Fig. S8 in Supplementary information. Results suggest that the models fit well and the loss does not correlate with window size, genre, or annotation layer.

Further, we capitalized on the adjustable window to control the length of context, ranging from 1 to maximum 6 words on both sides, constrained by the capacity of information processing by humans^94^. Considering the varying mean length of utterances in different corpora (Table S4 in Supplementary information), no window was presumably deemed optimal. Therefore, it is beneficial to examine the performances with a dynamic range of context. Table S5 in Supplementary information shows the amount of context within each window size in training across three genres. The differences between window sizes are similar across genres, except that there is more data for larger windows in adult conversation and written language than in child-directed speech.

#### Measure

To evaluate how the causative meaning was discriminated by the models, we retrieved prototypical causatives that commonly exist across human languages^73^ and non-causatives based on the definition proposed by Shibatani^74^. In particular, to reduce the impact of transitivity in the discrimination task, we included verbs that can be used intransitively in the category of causatives (e.g., “begin”, “open”) and transitive verbs in the category of non-causative (e.g., “like”, “take”). The selected verbs are listed below.

- Causatives (23): *begin, boil, break, burn, change, close, destroy, dry, fill, finish, freeze, gather, kill, lose, melt, open, raise, roll, sink, spread, stop, teach, turn*
- Non-causatives (9): *die, go, look, talk, want, like, think, say, take*

To check the semantic similarity between causatives and non-causatives, we employed the metric of cosine distance in a pairwise manner^17^. For each causative $i$ with the vector $\mathbf {c_{i}}$ each non-causative $\mathbf {n_{j}}$ in the set of non-causatives:

$$\begin{aligned} distance_{(i,j)} = 1 - cos(\theta _{(i,j)})=1-\frac{\mathbf {c_{i}}\cdot \mathbf {n_{j}}}{\Vert \mathbf {c_{i}}\Vert \Vert \mathbf {n_{j}}\Vert } \end{aligned}$$

Accordingly, the distance ranged from 0 to 2. For each model, the result included a list of 207 distance measures.

### Main studies and data analyses

In Study 1 we investigate the role of raw utterances in causative discrimination across different genres. Accordingly, only the lemmatized utterances were used as the training source for word embeddings. We trained embeddings for each window (from 1 to 6) with each genre (child-directed speech, adult conversation, written language), therefore obtaining 18 sets of pairwise cosine distances in the end. Since child-directed speech was set as the reference level, the baseline performance was calculated by averaging cosine distance of 10000 random pairs of frequent verbs in the embeddings models of child-directed speech, defined as appearing at least 10 times in each corpus. Subsequently, a Bayesian hierarchical model was built for distance scores within each window:

$$\begin{aligned} distance - baseline \sim genre + (1 + genre | caus ) + (1 + genre | noncaus ) \end{aligned}$$

Individual causative and non-causative verbs involved in the distance measures were treated as random factors, with both random intercept and random slope included. We examined the posterior distribution of the coefficient of “genre” to account for the effect of genre-specific information, and the posterior distribution of the intercept to assess the performance in child-directed speech compared to the respective baseline.

Study 2 examines the role of the additional layers “word class” and “syntax”. Like in Study 1 we measured the pairwise cosine distance between causatives and non-causatives in the embeddings models. For the baselines we calculated the mean distance of 10000 random pairs of frequent verbs for the models trained from raw utterances only. Hence, there were 18 baselines for 3 genres with their 6 windows respectively (see Fig. S9 in Supplementary information). For each genre within each window, a Bayesian hierarchical model was again built to examine the effect of syntactic layers on the cosine distance, i.e. the causative discrimination:

$$\begin{aligned} distance - baseline \sim layer + (1 + layer | caus ) + (1 + layer | noncaus ) \end{aligned}$$

The layer of raw utterances was set as the reference, and the coefficient of “layer” thus suggests the effect of syntactic information on causative discrimination.

In all models, the priors of both the intercept and the coefficient were set as a weakly informative Student-*t* distribution with location 0, degree of freedom 5, and the default scales as in the R package rstanarm^95^ (10 for intercepts and 2.5 for coefficients). Student-*t* distribution allows thicker tails to reduce the bias around 0. All models were fitted in Stan with help of rstanarm.

For model comparison we used Pareto-smoothed importance sampling as an efficient approximation of leave-one-out cross-validation and report bootstrapped Akaike weights (“Pseudo-BMA+”^76^). The results are shown in Fig. S2 in Supplementary information.

In addition, to take into consideration the uncertainty of the baselines (see Fig. S9 in Supplementary information), we reanalyzed the data with models that account for the measurement error of the dependent variable (see Sect. S4 and Figs. S10 and S11 for more detail).

### Probing distributional features in verbal constructions

We conducted three analyses to shed light on the distributional features in verbal constructions in different genres: (i) the average proportion of nominals in verbal constructions, (ii) the Shannon entropy^96^ of these nominals, and (iii) the entropy of verbal constructions per window size in the form of word-class tags (e.g. “NOUN verb NOUN” with window 1). For each genre with each window size, we bootstrapped 1000 verbal constructions for analyses (i) and (iii) and 1000 nominals for analysis (iii) to compute the respective measure, with the iteration of 1000.

## Supplementary Information

Supplementary Information.

## Data Availability

Data and codes can be accessed https://osf.io/hcj7y/?view_only=87bf26f2343c4a9ebc93d69aaaf6eddb.
