# Supplementary material for: Child-directed speech is optimized for syntax-free semantic inference
Source: Sci Rep. 2021 Aug 16;11:16527. doi: 10.1038/s41598-021-95392-x (PMC8368066; doi:10.1038/s41598-021-95392-x)
Supplement: Supplementary file 1 — Supplementary Information. [file 41598_2021_95392_MOESM1_ESM.pdf]

Child-directed speech is optimized for syntax-free semantic inference

Guanghao You, Balthasar Bickel, Moritz M. Daum, Sabine Stoll

Supplementary information

Supplementary analyses S1 to S4
Tables S1 to S5
Figs. S1 to S11

Data and codes can be accessed here.

Supplementary analyses

S1. Clustering using neighbor joining

In order to examine what semantics might be inferred from the Word2Vec models, especially the crucial model trained on raw utterances in child-directed speech with window 1, we performed clustering analysis with neighbor joining [1]. Simply put, neighbor joining is a bottom-up clustering algorithm that iteratively joins the pair of nodes that render the shortest distance. In our analysis, the distance matrix was calculated with pairwise cosine distances for all the 473 frequent verbs.

The analysis results for the crucial model exhibited clusters that were related to the semantics of causality (see the full plot of clusters in Fig. S3; Visualization of both Fig. S3 and Fig. S4 was rendered by ETE3 [2]). For examples, Fig. S4 shows a cluster of words closely related to causal meanings (e.g., *feed* "cause to eat", *tighten* "cause to be tight"), even including some important prototypical causatives (e.g., *open*, *spread*, and *change*), while excluding all the non-causatives involved in our main analyses. This strongly indicates that semantic inference does take place to some extent, thus aiding the discrimination between causatives and non-causatives.

S2. Additional test for syntactic information with lexical words

In addition to the two syntactic layers (i.e. "word class" and "syntax"), we tested for the effect of syntactic information from dependencies on a more basic level. Instead of using word classes in the representations of the dependencies we used lexical words from the layer "lexicon" (see Table S3). The idea was to investigate a layer that implicitly includes syntactic information relying on a more accessible form, i.e. individual words themselves (lexical forms). Thus, the generalization of word forms into word classes is not used in this layer. We added this layer to simulate a situation in which children have not yet generalized the dependency relations. To examine the effect of this layer on causative discrimination, we followed the same procedures as in Study 2.

As shown in Fig. S5, performance with this additional layer shows no clear improvement across all windows, but it tends to shift to the positive when the window size is small. By contrast, for written language, adding this layer is detrimental with a small window, yielding a negative effect [window 1: 95% CI (-0.0272, -0.0005); window 2: 95% CI (-0.0269, -0.0020)]. A possible reason is that long utterances with sophisticated or long dependencies make up a large proportion in written language, so that subtrees with lexical words could introduce a number of new contextual patterns with rich lexical information. Thus, generalization of these new patterns together with the original contexts is much more difficult than in child-directed speech, where both the complexity of dependency structure and the size of vocabulary are relatively low. Meaning

discrimination via distributional learning in adult conversation and written language hence shows no sign of profiting from implicit syntactic indications, but rather benefits from explicit structural tags such as word classes and dependency relation tags. This distinction is in line with the findings in Study 2, suggesting that raw lexical items in child-directed speech can be reliable sources for meaning discrimination, whereas explicitly-marked syntax is required in adult conversation and written language to discriminate between causatives and noncausatives.

S3. Additional test using annotations from the Manchester Corpus

We extracted lemmas, word-class tagging and dependency tagging information from the Manchester corpus to constitute the training sources for `Word2Vec` models. We applied the same modeling procedure as in main Study 2. The results (see Fig. S6) are in line with those using automatic parsing (see Fig. 2B), and meaning discrimination with raw utterances only in window 1 and 2 shows an even clearer effect compared with the baseline performance [window 1: 95% CI (0.0032, 0.0473); window 2: 90% CI (0.0019, 0.0369)]. Both word-class and dependency tagging do not exhibit facilitation in differentiating causative meaning.

S4. Regression analysis with baseline variance

In an additional test, we included the variance of the baselines (see Fig. S9) to run the regression described in Study 1 & 2. That is, the variance was considered as the error of the dependent variable in both regression analyses. This was implemented with the *mi()* syntax in the R package `brms` [3, 4]. The aim was to incorporate the uncertainty of the distance measure, as the variance could be large enough to impact the effects attested in both studies. A few models did not converge well with a gaussian distribution. Therefore, we transformed the data and modeled with a beta response distribution, which generally rendered good convergence and posterior predictive checks.

Fig. S10 and Fig. S11 show the results of the reanalyses. The effects are similar to what has been discovered in Study 1 & 2, but the confidence intervals of the coefficients are all widened, which is expected due to the added uncertainty. Nonetheless, raw utterances are still facilitative in child-directed speech, with the causative discrimination generally exhibiting above baseline performance (94.4% of the posterior samples of the intercept are above 0; see Fig. S10). Also, the coefficient of syntax stays positive for both adult conversation and written language (0 is outside most 90% CIs when the window size is larger than 1; see Fig. S11), despite showing a less prominent effect than the original analysis in Study 2. Besides, both syntax and word class exhibit no facilitation for causative discrimination in child-directed speech (0 is included in all 80% CIs and most 50% CIs), as we have found in Study 2.

References

[1] Saitou, N. & Nei, M. The neighbor-joining method: a new method for reconstructing phylogenetic trees. *Molecular biology and evolution* **4**, 406–425 (1987).

[2] Huerta-Cepas, J., Serra, F. & Bork, P. ETE 3: Reconstruction, Analysis, and Visualization of Phylogenomic Data. *Molecular Biology and Evolution* **33**, 1635–1638 (2016). URL `https://doi.org/10.1093/molbev/msw046`. `https://academic.oup.com/mbe/article-pdf/33/6/1635/7953632/msw046.pdf`.

[3] Bürkner, P.-C. brms: An R package for Bayesian multilevel models using Stan. *Journal of Statistical Software* **80**, 1–28 (2017).

[4] Bürkner, P.-C. Advanced Bayesian multilevel modeling with the R package brms. *The R Journal* **10**, 395–411 (2018).

Tables

Table S1: **Summary of the size of the original corpora**

Genre	Utterances	Tokens
Child-directed speech	696,125	2,921,818
Adult conversation	938,211	8,840,887
Written language	4,982,833	87,877,727

Table S2: **Summary of the size of the sampled corpora.** For the column of frequent verbs, the number of verbs that occur no less than 10 times is reported.

Genre	Sessions	Utterances	Tokens	Frequent verbs
Child-directed speech	793	696,125	2,921,818	601
Adult conversation	255	326,359	3,227,762	1,340
Written language	111	189,180	3,251,562	2,325

Table S3: **Example of flattened subtrees extracted from dependencies for layer "syntax" and layer "lexicon" for the example utterance "John says Mary believes Tom broke the windows" in Fig. 2A, where the arrows are directed from the head to its dependents in each subtree**. Here "nsubj" denotes "nominal subject", "dobj" denotes "direct object", and "det" denotes "determiner". Asterisks are used with the tags to distinguish themselves from words.

syntax	lexicon
*nsubj say believe	john say believe
*nsubj believe break	mary believe break
*nsubj break *dobj	tom break window
*det *dobj	the window

Table S4: **Number of utterances with different lengths in each corpus (in words)**

Length	Child-directed speech	Adult conversation	Written language
1	180,860	60,895	5,043
2	75,287	27,825	7,432
3	89,529	25,460	7,185
4	83,597	23,996	6,788
5	71,007	22,064	7,213
6	55,010	19,579	6,852
7	41,893	17,055	6,935
8	31,110	14,428	6,710
9	21,725	12,375	6,599
10	14,729	10,521	6,366
11	9,992	8,741	6,336
12	6,577	7,640	6,213
13	4,366	6,722	6,051
>13	10,443	69,058	103,457
Mean length	4.19	9.89	17.19

Table S5: **Number of contextual items included within each window in the training of raw utterances**

Window	Child-directed speech	Adult conversation	Written language
1	7,235,886	7,108,242	6,881,484
2	13,079,522	13,563,766	13,384,608
3	17,530,908	19,366,572	19,509,372
4	20,951,764	24,638,450	25,265,862
5	23,492,664	29,435,050	30,668,942
6	25,332,666	33,807,292	35,732,982

Figures

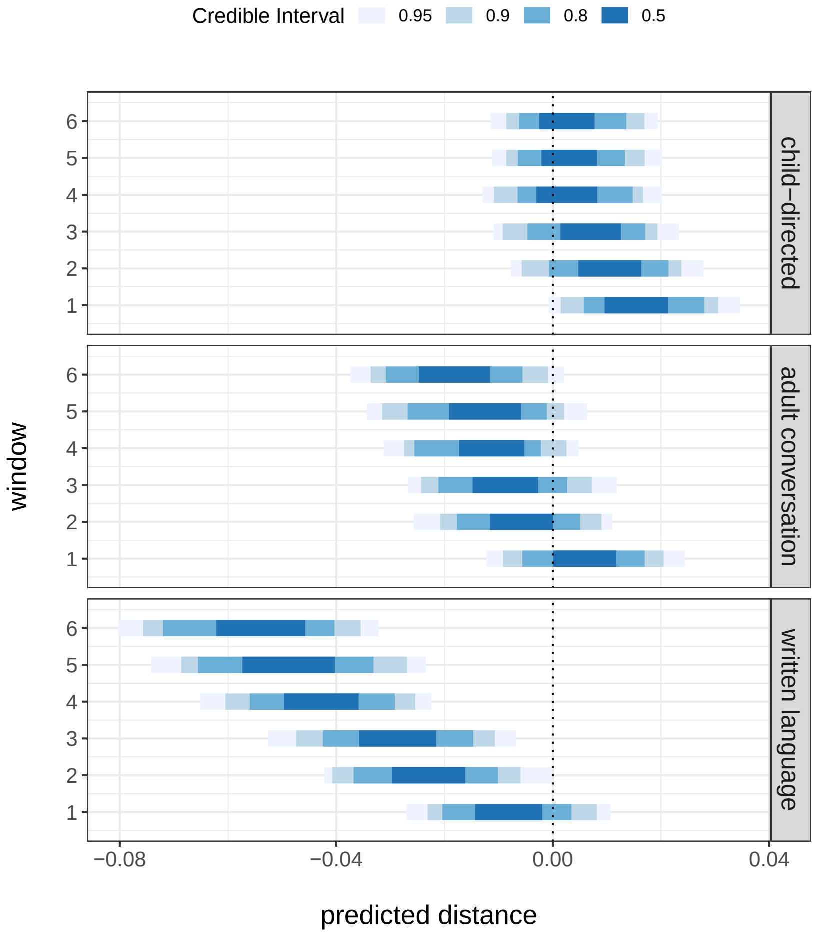

Figure S1: **Above-baseline predicted cosine distance between causative and non-causative meanings for each genre in Study 1.** Child-directed speech shows a major shift to the positive side, with window 1 achieving the best above-baseline performance. The other two genres mostly perform under the baseline.

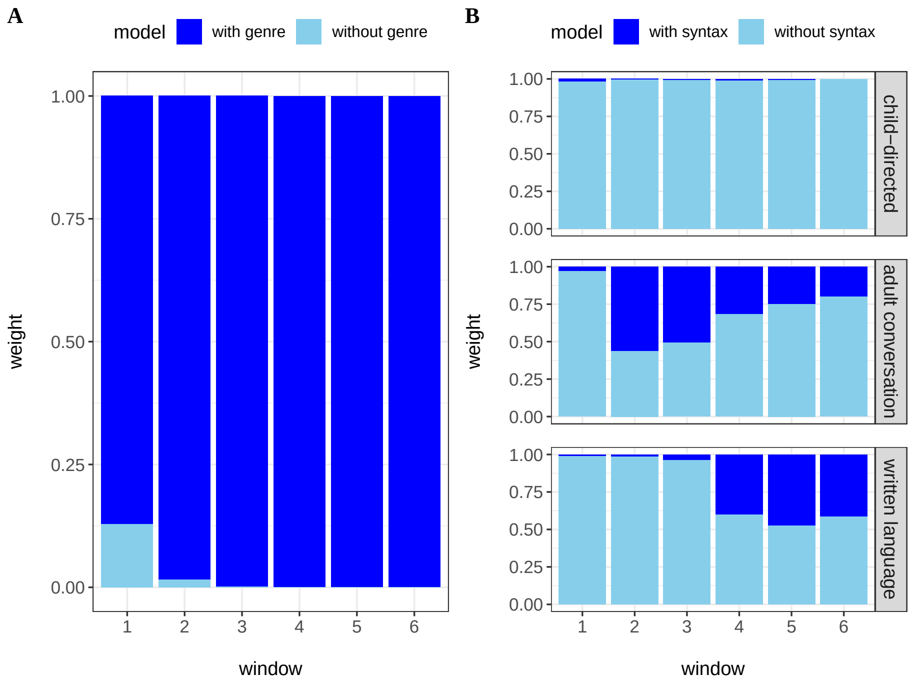

Figure S2: **Akaike weights of predictive performance in leave-one-out cross-validation (using Pseudo-BMA+) in Study 1 (A) and Study 2 (B).** Models without genre as a predictor gain negligible weight in Study 1. In Study 2, the full model with syntax in both adult conversation and written language leverages noticeably more weight than in child-directed speech when the window is larger than 1.

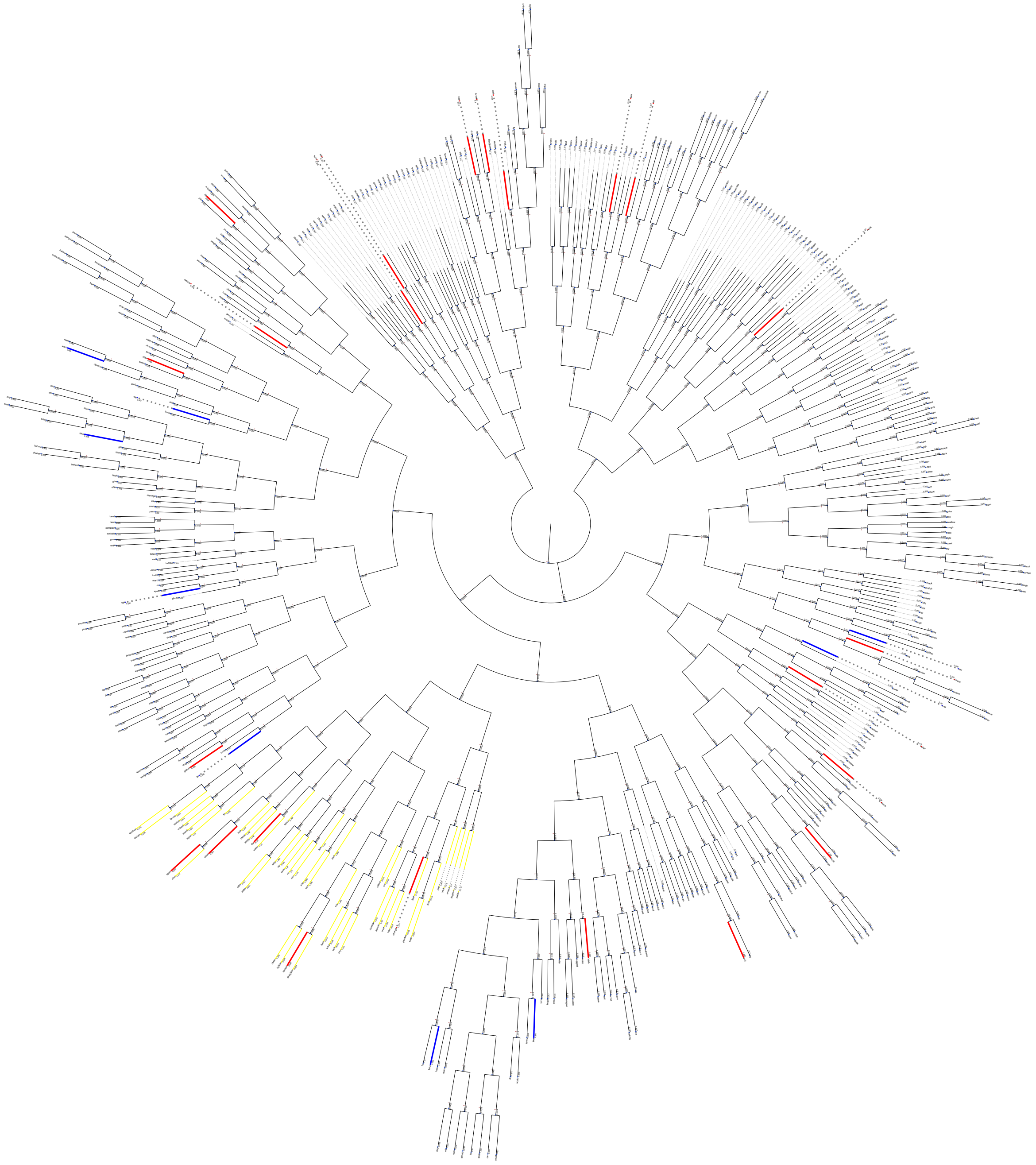

Figure S3: **Results of neighbor-joining analysis of words for the model trained on raw utterances in child-directed speech with window 1, based on their pairwise cosine distances in the vector space** All frequent 473 verbs are plotted. Leaf branches are marked for several categories: 23 prototypical causatives are marked in red, 9 non-causatives in blue, and an example cluster of words associated with causal semantics is marked in yellow. This example cluster is further plotted in Fig. S4. Distances are marked on the branches.

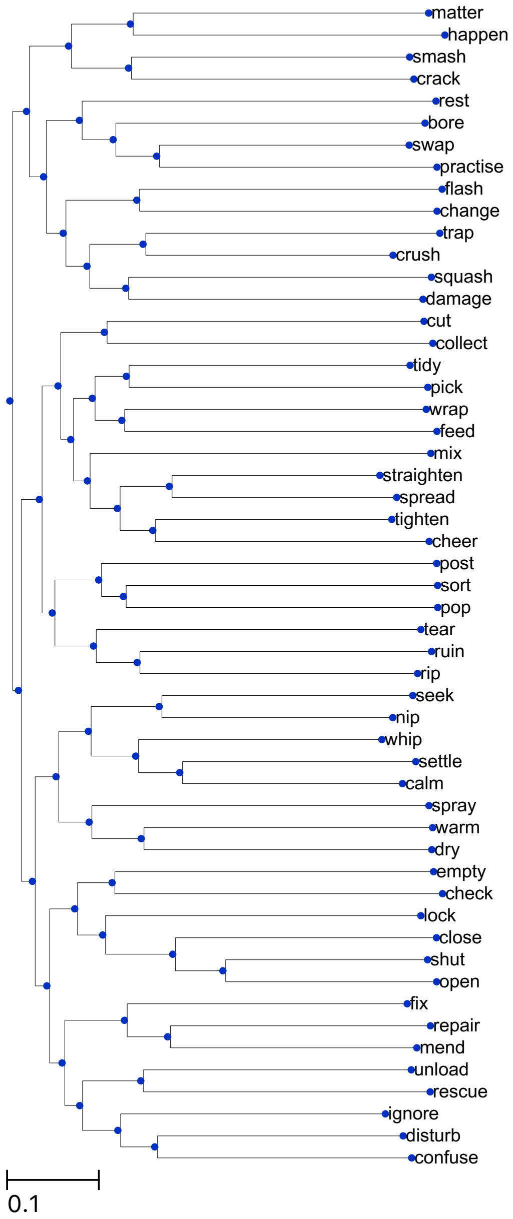

Figure S4: **Example cluster of words for the model trained on raw utterances in child-directed speech with window 1, based on their pairwise cosine distances in the vector space** 53 verbs included in this cluster. The lengths of branches represent the cosine distances between nodes. These verbs clearly convey the shared semantics of causality.

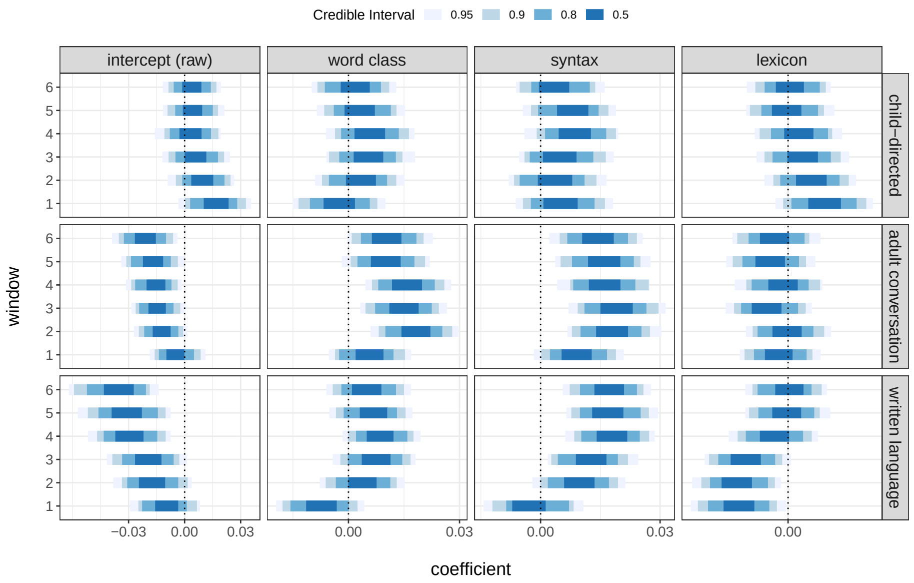

Figure S5: **The effect of layer "lexicon" on causative discrimination.** Child-directed speech tends to benefit from the additional layer of syntactic information in the form of lexical items. No such improvement is seen in either written language or adult conversation, with written language particularly showing detrimental effect by this layer when the window size is smaller than 4 (95% CI).

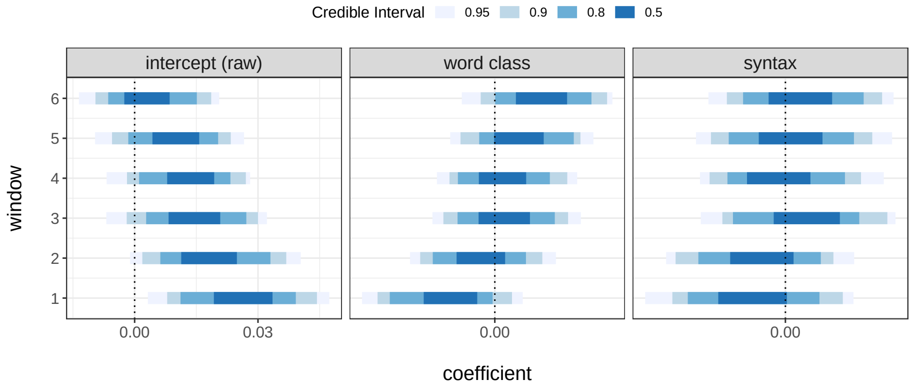

Figure S6: **The effects of different information layers on causative discrimination in child-directed speech using hand-tagged annotations from the Manchester corpus** The `word2vec` model trained from raw utterances exhibits clear above-baseline performance with window 1 (95% CI) and window 2 (90% CI), whereas additional syntactic information yields no improvement for causative discrimination. These results confirm those trained from automatically-parsed annotations.

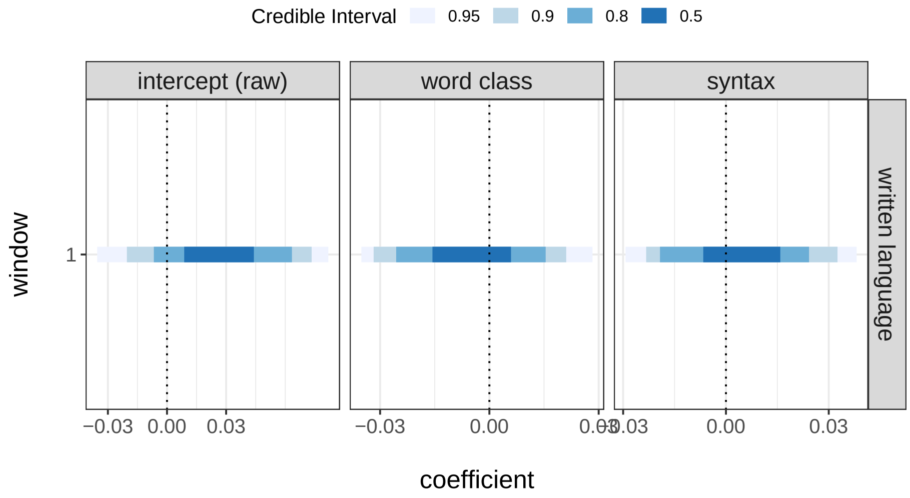

Figure S7: **Results of layer effect for the embeddings model with 50 dimensions for child-directed speech with window 1.** The causal inference is harmed by setting a low number of dimensions, while syntactic layers show no facilitation in causal inference.

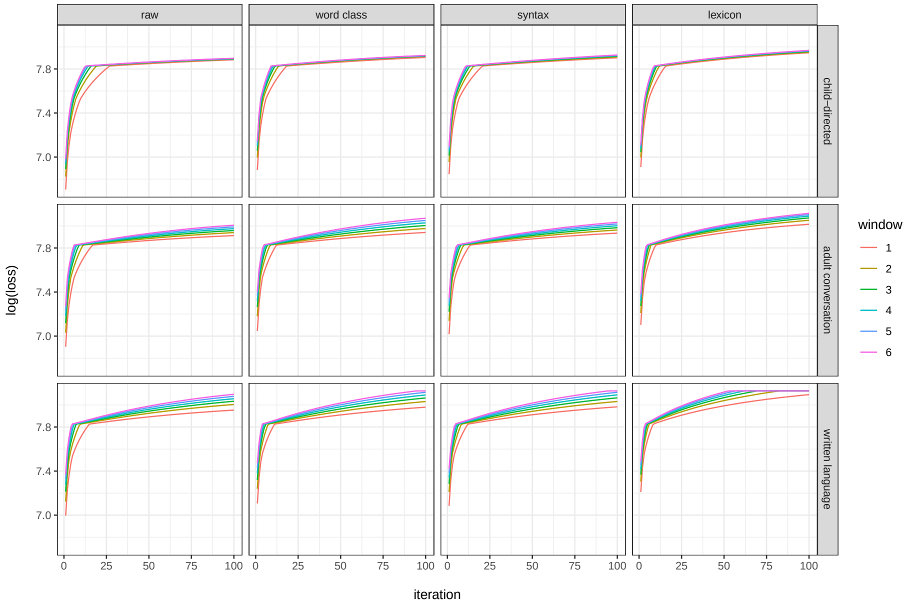

Figure S8: **Loss after each iteration of training for each model.** Due to the large value, the loss measure is shown on a $\log_{10}$ scale. In each model, there is a clear starting point of convergence, after which the loss increases only slowly. No major difference is shown across windows, genres and annotation layers. all models show stabilized training loss after a few iterations, thus suggesting good model fitting.

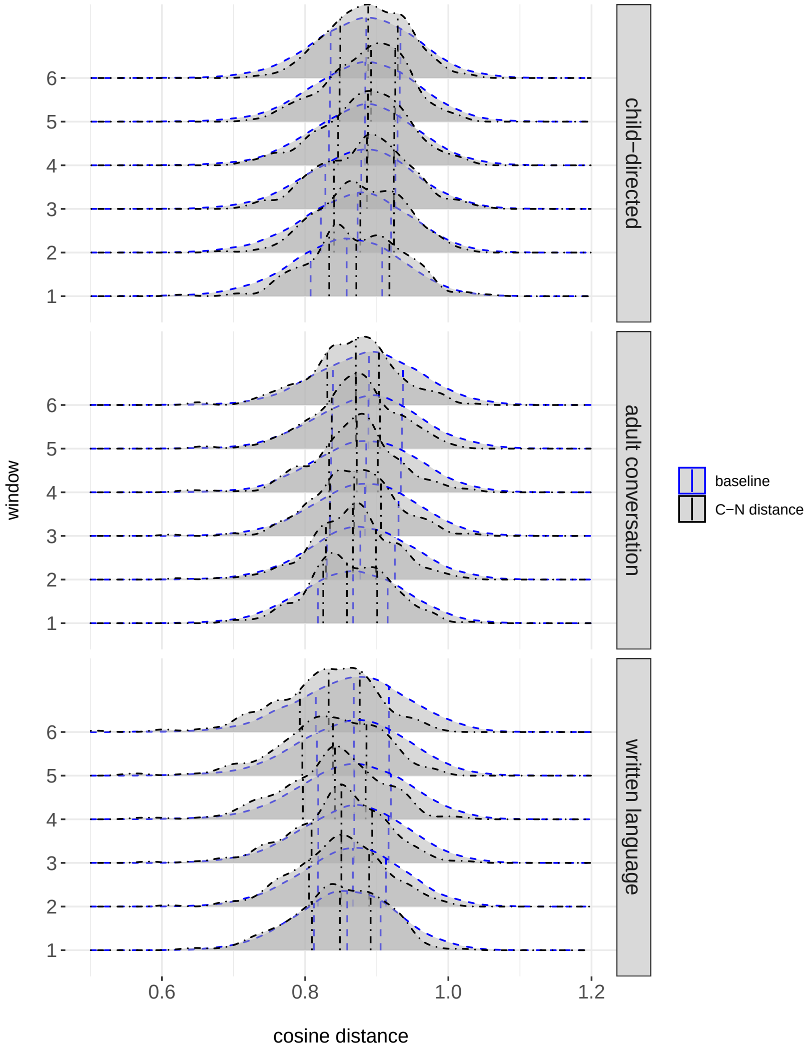

Figure S9: **Distribution of cosine distances between random verbs in each word embeddings model for setting the baseline, and distribution of cosine distances between causative-noncausative (C-N) pairs** 10,000 random verb pairs were sampled for each model. (27 outlier samples are omitted from the plot). The C-N distances are from the 18 models with raw utterances. Vertical lines mark the quantiles of each distribution. C-N distances generally shift to the right in child-directed speech, while shifting to the left in the other two genres.

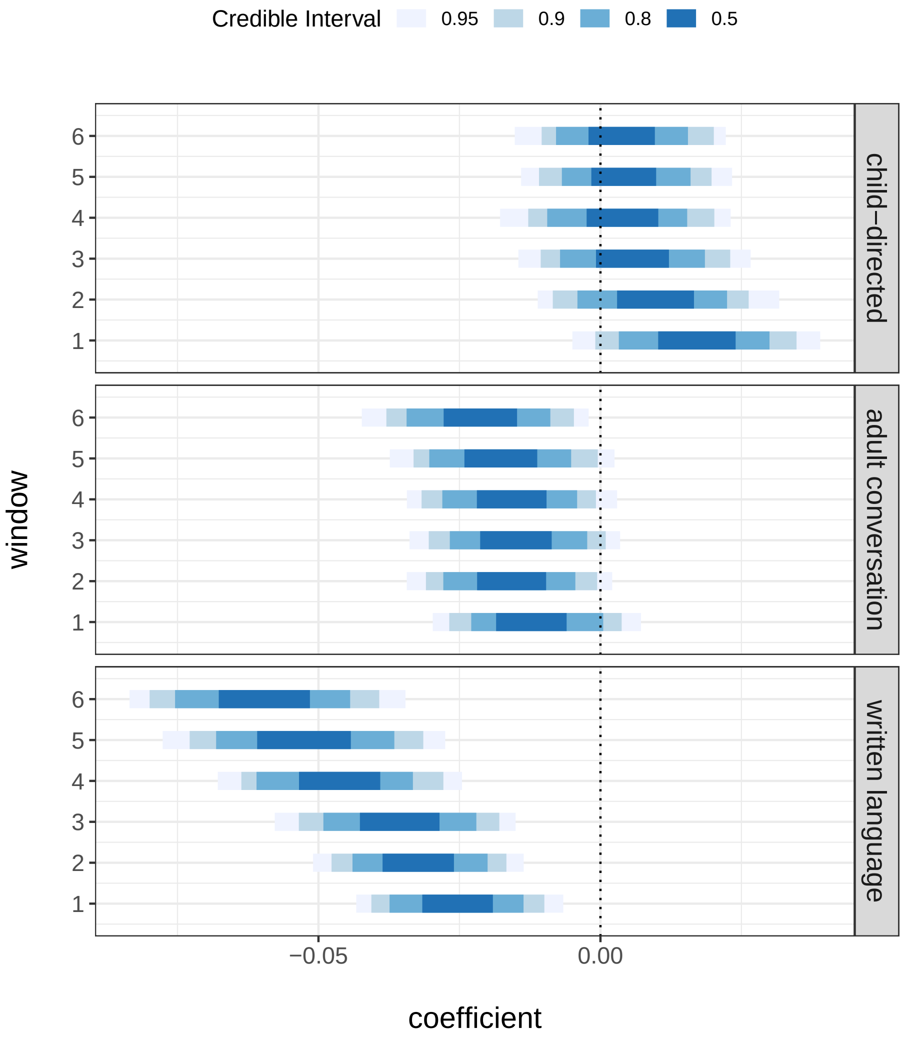

Figure S10: **Reanalysis of Study 1 with baseline variance.** Difference of performance is still retained when baseline variance is included as the error of the distance in the regression, although the effect is slightly diminished. Credible intervals represent highest posterior densities.

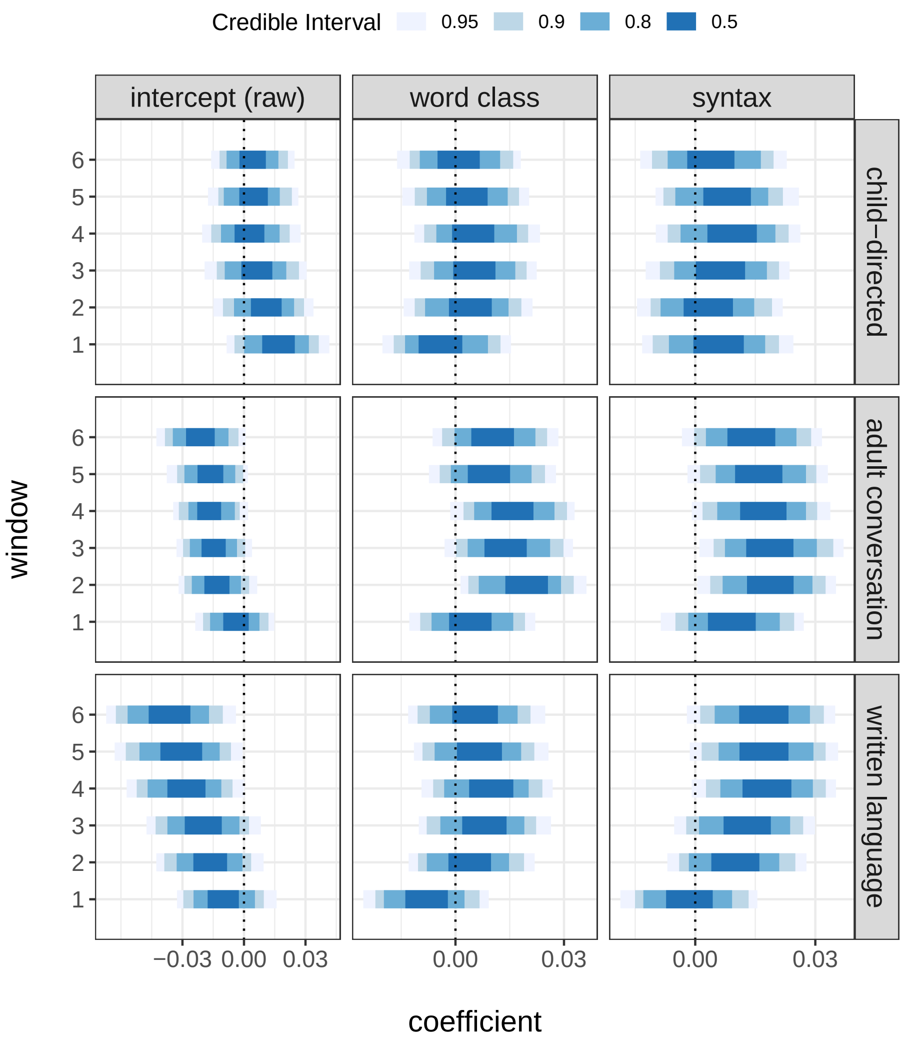

Figure S11: **Reanalysis of Study 2 with baseline variance.** When baseline variance is included as the error of the distance in the regression, syntax and word class are still not facilitative in child-directed speech, whereas these additional layers aid the causative discrimination in adult conversation and written language. Credible intervals represent highest posterior densities.
